# Proliferative glomerulonephritis with monoclonal immunoglobulin IgG1-κ deposition in a patient with type 1 diabetes: a case report and literature review

**DOI:** 10.3389/fmed.2026.1799381

**Published:** 2026-06-15

**Authors:** Xi Li, Xiaoyan Zhang, Xinjun Dai, Yan Zhang, Tian Lan, Jian Li, Minghui Wu, Tianhang Jia, Jie Li

**Affiliations:** 1 Heping Hospital Affiliated to Changzhi Medical College, Changzhi, China; 2 Liuyang Hospital of Traditional Chinese Medicine, The Second Western and Chinese Medicine Hospital of Hunan University of Chinese Medicine, Changsha, China; 3 The Third People’s Hospital, Taiyuan, China; 4 Xi’an First Hospital, Xi’an, China

**Keywords:** PGNMID, proliferative glomerulonephritis with monoclonal IgG1-κ deposits, MGRS, cyclophosphamide, infection, type 1 diabetes mellitus

## Abstract

**Background:**

Proliferative glomerulonephritis with monoclonal immunoglobulin deposits (PGNMID) is a rare glomerular disease characterized by edema, proteinuria, and renal insufficiency. No established treatment protocol is currently available. We report a case of PGNMID with monoclonal IgG1-κ deposits in a patient with a 19-year history of type 1 diabetes.

**Case presentation:**

A 30-year-old woman presented with a 1-week history of nausea, vomiting, and generalized edema. She had a 19-year history of type 1 diabetes mellitus with poor glycemic control. Three months before admission, she had been hospitalized for fever and was diagnosed with a urinary tract infection, during which her renal function was normal. After discharge, she continued to experience recurrent episodes of fever. One week before admission, she gradually developed generalized edema accompanied by decreased urine output. On admission, she was found to have nephrotic-range proteinuria, hypoalbuminemia, anemia, acute renal failure, and acute heart failure. After antimicrobial therapy, renal replacement therapy, and nutritional support, the patient underwent renal biopsy. Immunofluorescence showed diffuse, global, granular, and lumpy deposits of IgG, C3, and κ light chain along the glomerular capillary loops and in the mesangial regions, with strong IgG1 positivity. Electron microscopy revealed proliferation of glomerular capillary endothelial cells, accompanied by segmental mesangial matrix interposition into the basement membrane, resulting in a double-contour appearance. Segmental hump-like subepithelial electron-dense deposits were also observed. No abnormal monoclonal bands were detected by serum or urine immunofixation electrophoresis, and the serum and urine free light chain κ/λ ratios were within the normal range. Bone marrow aspiration revealed 0.5% plasma cells. The final diagnosis was proliferative glomerulonephritis with monoclonal IgG1-κ deposits (IgG1κ-PGNMID). The patient was treated with prednisone, cyclophosphamide, and renal replacement therapy, with a good initial response. She was temporarily weaned from renal replacement therapy in the short term, but subsequently required long-term maintenance dialysis.

**Conclusion:**

In patients with a long-standing history of diabetes, the presence of a preceding infection, hypocomplementemia, nephrotic-range proteinuria, and rapidly progressive renal dysfunction should prompt suspicion for non-diabetic kidney disease. In such cases, renal biopsy, together with serum and urine immunofixation electrophoresis, free light chain assays, complement testing, and rheumatologic autoantibody screening, is essential for the diagnosis and differential diagnosis of PGNMID and related disorders. For patients with confirmed PGNMID who lack clear evidence of monoclonality or decline targeted therapy, prednisone combined with cyclophosphamide may represent an effective alternative treatment option.

## Introduction

Proliferative glomerulonephritis with monoclonal immunoglobulin deposits (PGNMID) is a rare type of monoclonal gammopathy of renal significance (MGRS). Detection rate by biopsy is approximately 0.17%–3.7% (1). Pathogenesis is that monoclonal immunoglobulins deposited in the mesangial area of the glomeruli and capillary walls cause complement activation, glomerular inflammation and hyperplasia. The main antibody involved in the deposits is IgG. Among them, IgG3 is the most common subtype, accounting for about 60%–68% (2). The IgG1 subtype accounts for 24%–29% of the detected cases. This subtype is more likely to manifest as nephrotic syndrome and monoclonal immunoglobulin is more frequently detected in the blood and bone marrow. Meanwhile, it is more often accompanied by extrarenal diseases (3, 4).

The clinical manifestations of PGNMID are non-specific, including edema, hypertension, renal insufficiency, proteinuria, hematuria. Elderly are the main patients. Non-specific clinical manifestations make diagnosis difficult, especially in patients with extrarenal disease, which can only be definitively diagnosed by kidney biopsy (3). Urinary protein reduction, immunosuppression and renal replacement therapy are the main treatment methods in the past. The current treatment trend is to carry out chemotherapy and targeted therapy for abnormal monoclonal immunoglobulin that produces nephrotoxicity. Common drugs include bortezomib, rituximab, daratumumab (5, 6).

In this context, we report a case of a young female patient who presented with recurrent infections followed by nephrotic syndrome and acute renal failure. No abnormal monoclonal immunoglobulin was detected in her serum or urine. A renal biopsy confirmed the diagnosis of proliferative glomerulonephritis with monoclonal IgG1-κ deposits. The patient responded well to combination therapy with prednisone and cyclophosphamide, which may provide a valuable reference for future clinical studies

## Case presentation

A 30-year-old Chinese woman was admitted for nausea, vomiting, and generalized edema for 1 week. Three months before the current admission, she developed fatigue, poor appetite, and fever (peak temperature 39.0°C) and was hospitalized at our institution. Initial laboratory tests showed proteinuria (1+), urinary white blood cells of 837/μL, a urinary albumin-to-creatinine ratio (UACR) of 58.66 mg/g, and normal renal function, with a urea level of 4.67 mmol/L (reference range, 2.8–7.6 mmol/L) and a serum creatinine level of 72 μmol/L (reference range, 41–73 μmol/L). Both blood and urine cultures were positive for Escherichia coli. She was diagnosed with acute pyelonephritis with bacteremia and treated with intravenous levofloxacin 0.5 g once daily. After 1 week of treatment, repeat testing showed resolution of proteinuria and a decrease in urinary white blood cells to 88/μL. She was then discharged and continued oral levofloxacin 0.5 g once daily for an additional week. Four weeks before the current admission, the patient developed intermittent fever (37.5–38.3°C) and occasional hypoglycemia, accompanied by rhinorrhea and sore throat. She denied cough, sputum production, urinary frequency, urgency, dysuria, diarrhea, myalgia, and arthralgia. She self-administered oral amoxicillin 250 mg every 8 h for 2 weeks, but her symptoms did not improve significantly. One week before admission, she developed nausea, vomiting, decreased urine output, and gradually progressive generalized edema. After 48 h of persistent oliguria (approximately 500 mL/24 h), she presented to our hospital for further evaluation.

Of notable relevance in the patient’s past medical history is a diagnosis of type 1 diabetes mellitus established 19 years ago, for which she initiated subcutaneous insulin therapy with aspart insulin (pre-meal) and detemir insulin (bedtime) at variable doses. However, the patient reported poor dietary compliance and consequently inadequate glycemic control. Over the past decade, she had been hospitalized multiple times for diabetic ketoacidosis. Throughout long-term follow-up and during previous hospitalizations, her renal function remained stable, with no proteinuria and normal UACR documented. She had no other significant long-term medication history.

She is a freelancer. She has no history of alcohol, tobacco or other substance use. Nor does she have a family history of cancer, autoimmune diseases, kidney diseases, gastrointestinal diseases, pulmonary diseases or heart diseases.

Physical examination on admission revealed a body temperature of 36.2°C, heart rate of 75 beats/min, respiratory rate of 20 breaths/min, and blood pressure of 161/99 mmHg. The patient appeared pale and had facial edema. No obvious dry or moist rales were heard on lung auscultation. The heart rhythm was regular. The abdomen was soft, without tenderness or rebound tenderness, and neither the liver nor the spleen was palpable below the costal margin. Mild pitting edema was present in both lower extremities. No superficial lymphadenopathy was detected. The remainder of the physical examination was unremarkable.

Laboratory tests performed urgently on admission are summarized in Table 1. On hospital day 2, urine culture revealed Escherichia coli (bacterial count > 1 × 10^5^ CFU/mL; susceptibility to piperacillin/tazobactam: MIC ≤ 4 μg/mL). The patient denied cough or sputum production, so no sputum sample was collected for culture.

**TABLE 1:** Clinical and laboratory examination results at admission.

Laboratory test	On admission	Reference range
White blood cell (WBC)	8.1	(3.5–9.5) 109/L
Hemoglobin (Hb)	76	(3.8–5.1) g/L
Platelet (PLT)	253	(125–350) 109/L
Neutrophil percentage (NEUT%)	0.818	0.40–0.75
Urine white blood cells	2,111	(0–9) per μL
Albumin (ALB)	26.2	(40–55) g/L
Glucose (GLU)	4.41	(3.89–6.11) mmol/L
Urea	30.81	(2.8–7.6) mmol/L
Creatinine (Crea)	347	(u41–73) umol/L
Bicarbonate (HCO_3_^-^)	14.9	(21–31) mmol/L
Potassium (K^+^)	6.13	3.5–5.3 mmol/L
C-reactive protein (CRP)	9.6	(0–5) mg/L
Procalcitonin (PCT)	0.5	(0–0.05) ng/ml
Hemoglobin A1c (HbA1c)	6.9	< 5.7%
NT-proBNP	6,805	(0–125) ng/L

**FIGURE 1:**
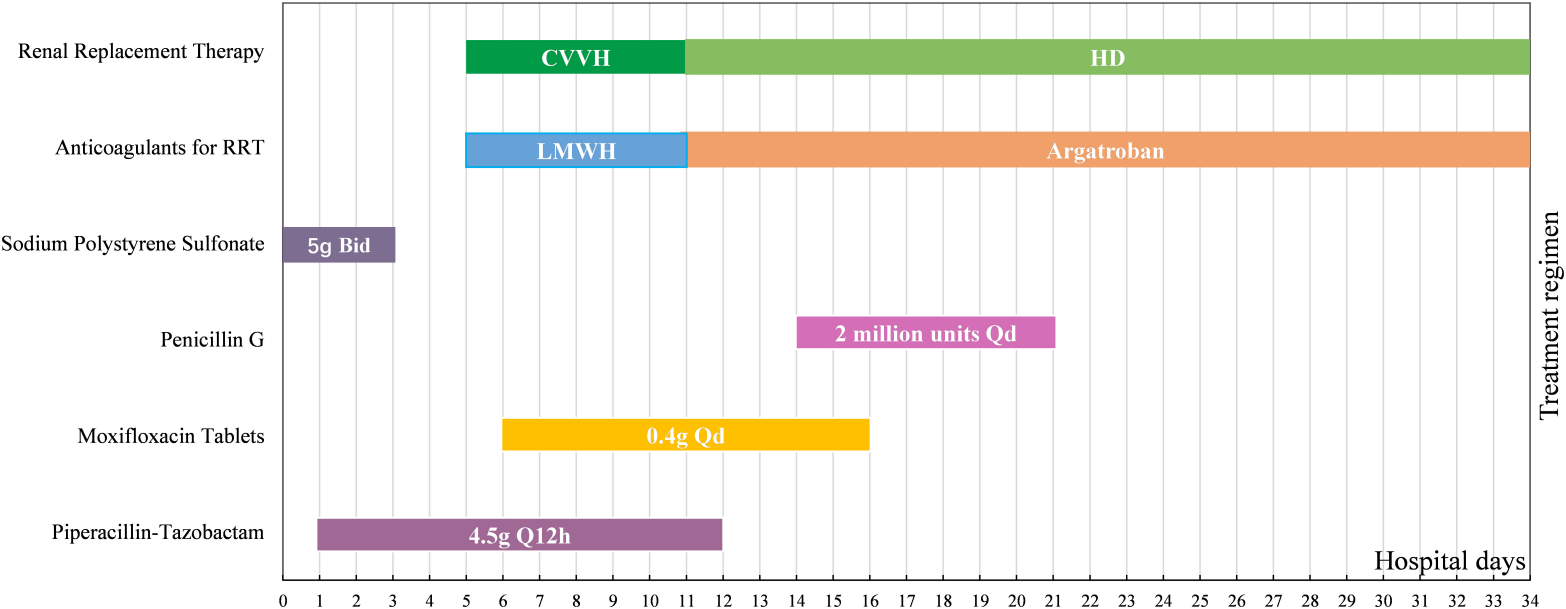
Temporal course of renal replacement therapy. CVVH, continuous venovenous hemofiltration; HD, hemodialysis; LMWH, low molecular weight heparin; 0, On the day of admission.

**FIGURE 2:**
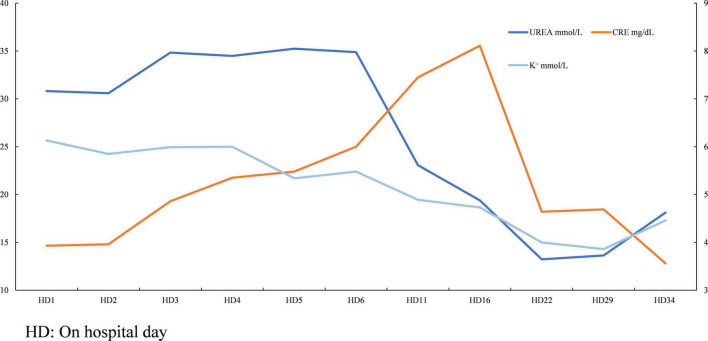
Timeline of changes in renal function parameters and serum potassium levels. HD1, On hospital day 1.

The patient was treated with piperacillin-tazobactam (4.5 g IV q12 h) for infection control and furosemide (40–80 mg/d IV push) for diuresis and hyperkalemia management (supplemented with oral cation-exchange resin; details in Figure 1). After five days of treatment, she continued to experience fatigue, with worsening generalized edema and new-onset cough, shortness of breath, and orthopnea. Daily urine output remained low, around 700–800 mL. Laboratory tests showed progressive renal function deterioration: urea increased to 35.24 mmol/L and serum creatinine to 484 μmol/L (compared to admission levels of 30.81 mmol/L and 347 μmol/L, respectively, Figure 2). Given that diabetic kidney disease (DKD) alone was considered insufficient to explain this rapid clinical progression, a coexisting non-diabetic kidney disease (NDKD) was strongly suspected. The patient was subsequently transferred to the nephrology department for renal replacement therapy and further etiological evaluation.

**FIGURE 3:**
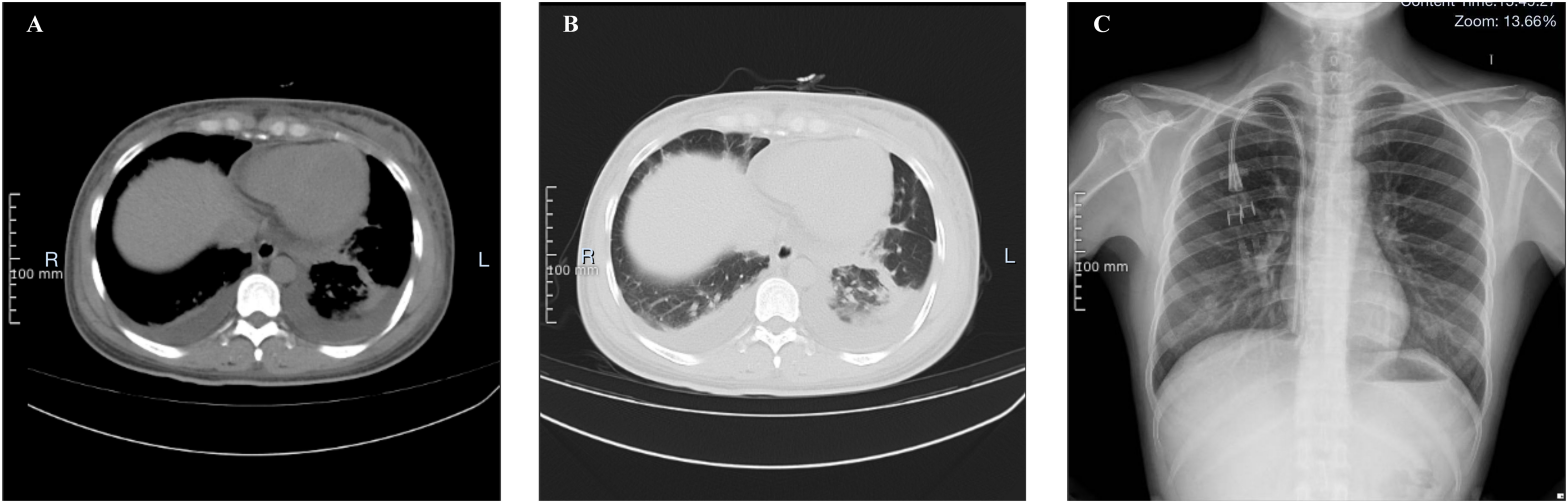
Chest imaging changes before and after treatment. **(A)** CT mediastinal window; **(B)** CT lung window; **(C)** chest radiograph.

After referral to the nephrology specialty, further laboratory tests were performed (Table 2): immunoglobulin IgG 16.1 g/L, complement C3 0.06 g/L, anti-streptolysin O 1,286.3 IU/mL; anti-MP antibody titer 1:160; immunoglobulin IgA, IgM, complement C4, antinuclear antibody (ANA), anti-double-stranded DNA (anti-dsDNA) antibody, ENA antibody panel, ANCA, anti-PLA2R antibody, and anti-GBM antibody were all within normal limits. Hepatitis B surface antigen (HBsAg), hepatitis C (HCV) antibody, Treponema pallidum antibody, human immunodeficiency virus (HIV) antibody, COVID-19 virus, influenza A virus antigen, influenza B virus antigen, Epstein-Barr virus, and respiratory syncytial virus were all negative. No abnormal monoclonal bands were detected on serum and urine immunofixation electrophoresis. Chest CT showed bilateral pulmonary inflammation with bilateral pleural effusion (Figures 3A, B). Abdominal ultrasound revealed increased renal parenchymal echogenicity in both kidneys (left kidney size approximately 13.2 cm × 5.2 cm). Cardiac ultrasound showed: ejection fraction 71%; small pericardial effusion; coordinated wall motion.

**TABLE 2:** Clinical and laboratory examination results when transferred to nephrology department.

Laboratory test	After transfer	Reference range
Immunoglobulin IgG	16.1	(7–16) g/L
Immunoglobulin IgA	2.66	(0.7–4.0) g/L
Immunoglobulin IgM	1.73	(0.4–2.3) g/L
Complement C3	0.06	(0.4–2.3) g/L
Complement C4	0.31	(0.10–0.40) g/L
Antistreptolysin O (ASO)	1286.3	< 200 IU/mL
anti-MP antibody titer	1:160	< 1:40

**FIGURE 4:**
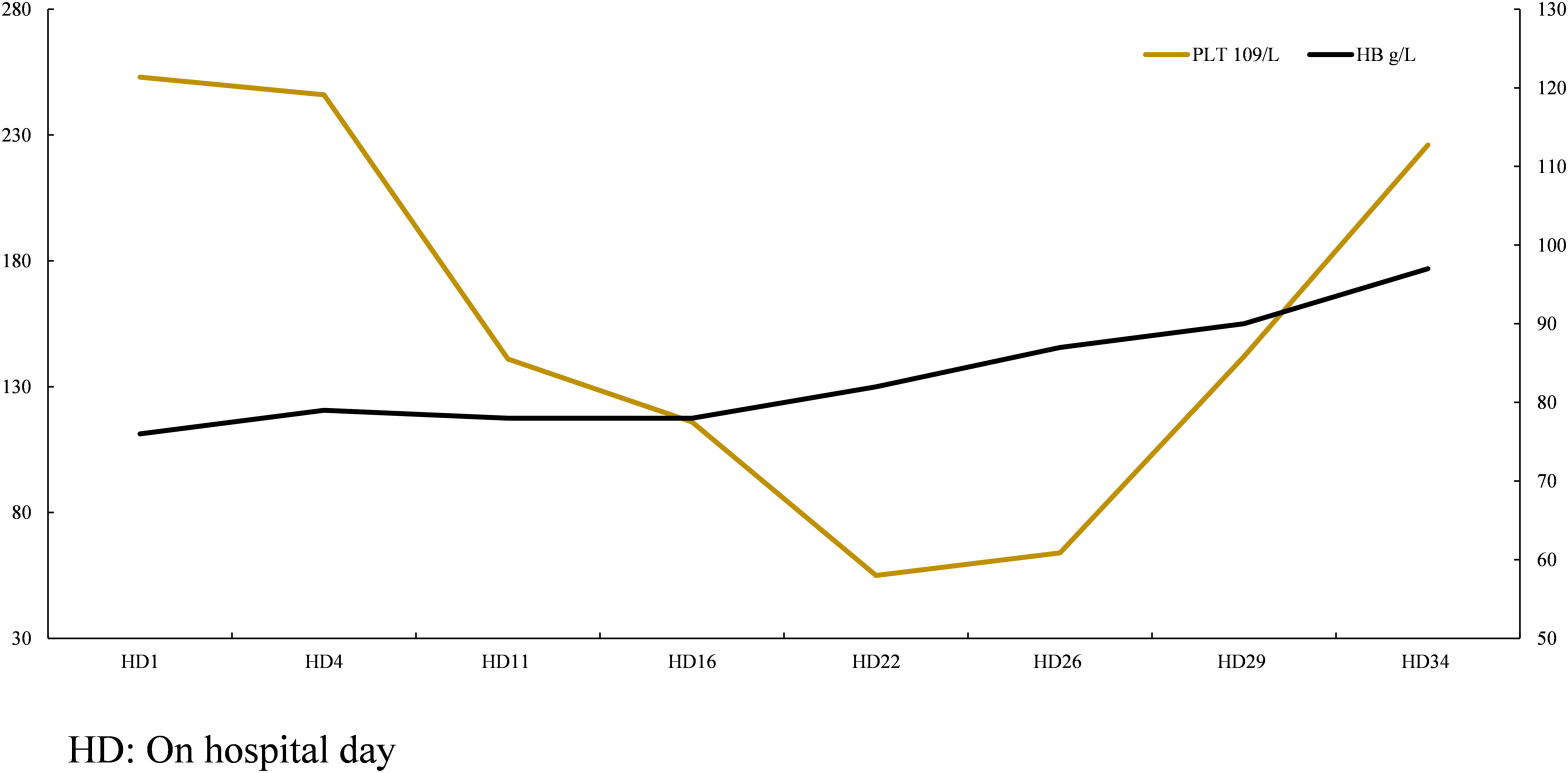
Temporal changes in hemoglobin and platelet levels during hospitalization. HD1, On hospital day 1.

The patient initiated active renal replacement therapy on the day of transfer (modality: CVVH, anticoagulated with low molecular weight heparin sodium, receiving intermittent bedside therapy for 5 days, followed by hemodialysis) (Figure 1). Due to a positive Mycoplasma pneumoniae result and a gradual rise in anti-streptolysin O to 1,396.3 IU/mL, treatment with “Moxifloxacin 0.4 g QD orally” and “Penicillin 2 million units IV QD” was sequentially added. On the sixth day of renal replacement therapy (RRT), a decrease in platelet count to 141 × 10^9^/L was observed (compared to a pre-RRT level of 246 × 10^9^/L), with a hemoglobin level of 78 g/L (versus 76 g/L on admission). Consequently, the dose of low molecular weight heparin sodium was reduced to 1,000 U. After four days of observation, a repeat test showed a further decline in platelets to 116 × 10^9^/L, with hemoglobin remaining at 78 g/L. The low molecular weight heparin sodium dose was again reduced, this time to 800 U. After five days of monitoring, the platelet count decreased to 55 × 10^9^/L, although hemoglobin had increased to 82 g/L and bilirubin levels were within the normal range. At this juncture, a diagnosis of “heparin-induced thrombocytopenia” was considered. Low molecular weight heparin was therefore discontinued and replaced with anticoagulation using “Argatroban 20 mg per dose.” After three days of observation, the platelet count rose to 64 × 10^9^/L, with hemoglobin level at 87 g/L (Figure 4). The patient showed improved mental status and appetite, with no cough, sputum production, or fever. Urine output was approximately 900 ml/24 h. Following transfusion of one unit of platelets, a renal biopsy was performed.

**FIGURE 5:**
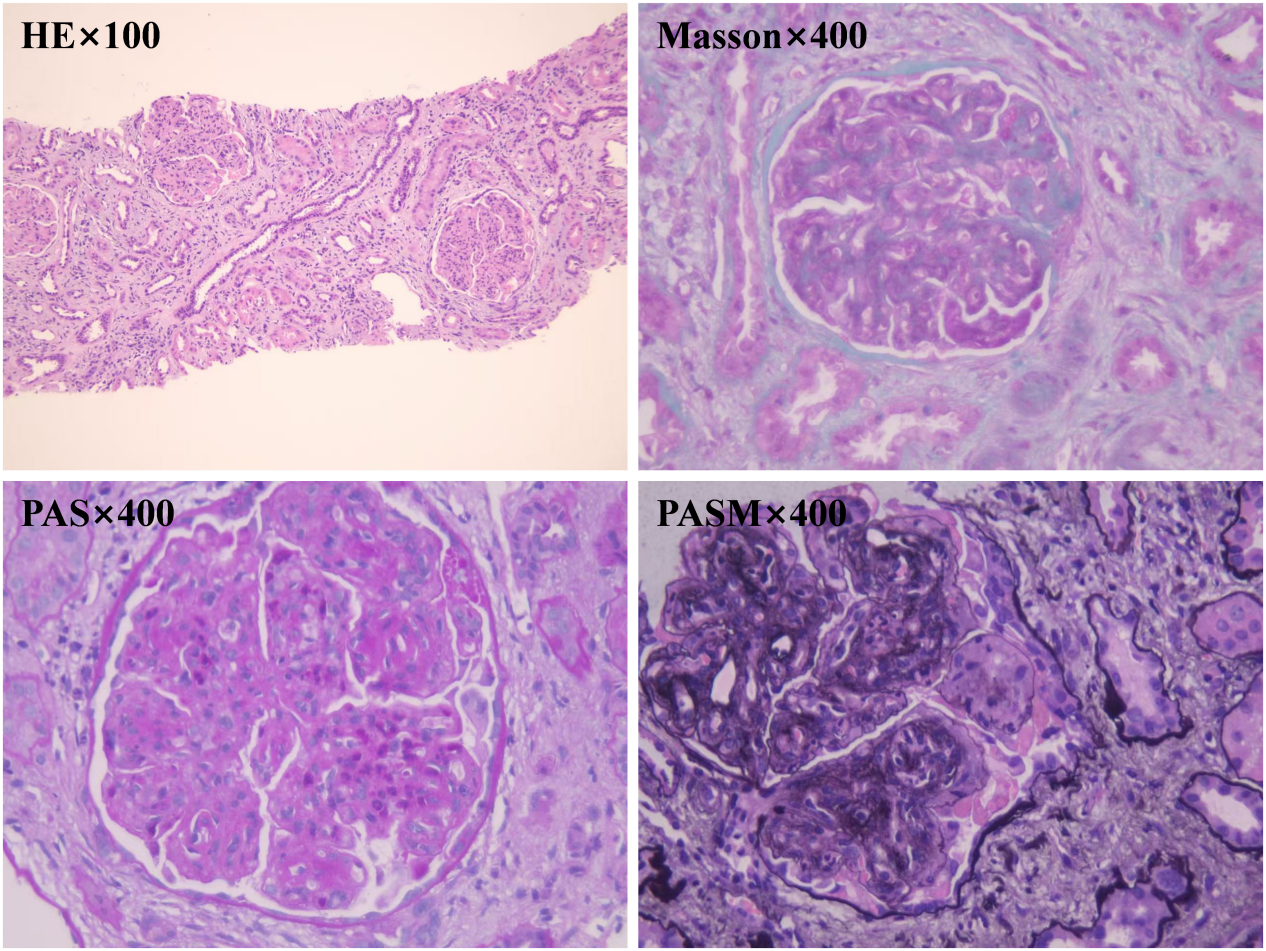
Light microscopy (LM) findings showing moderate-to-severe diffuse mesangial cell and matrix proliferation with diffuse glomerular basement membrane thickening and double contour formation.

**FIGURE 6:**
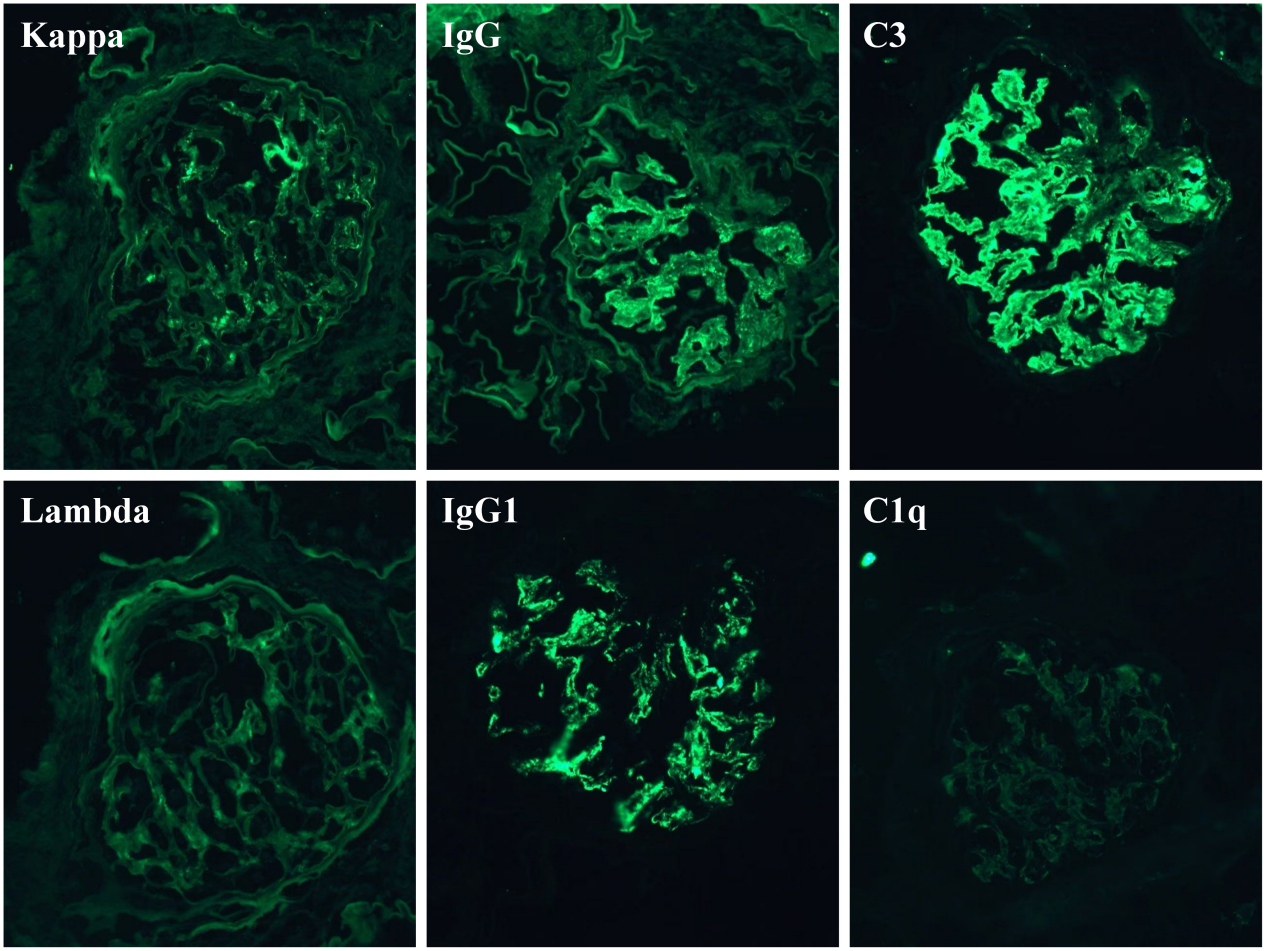
Immunofluorescence showing diffuse global deposition of IgG and C3 along mesangial areas and glomerular capillary loops. Staining is restricted to a single IgG1 subclass and κ light chain.

**FIGURE 7:**
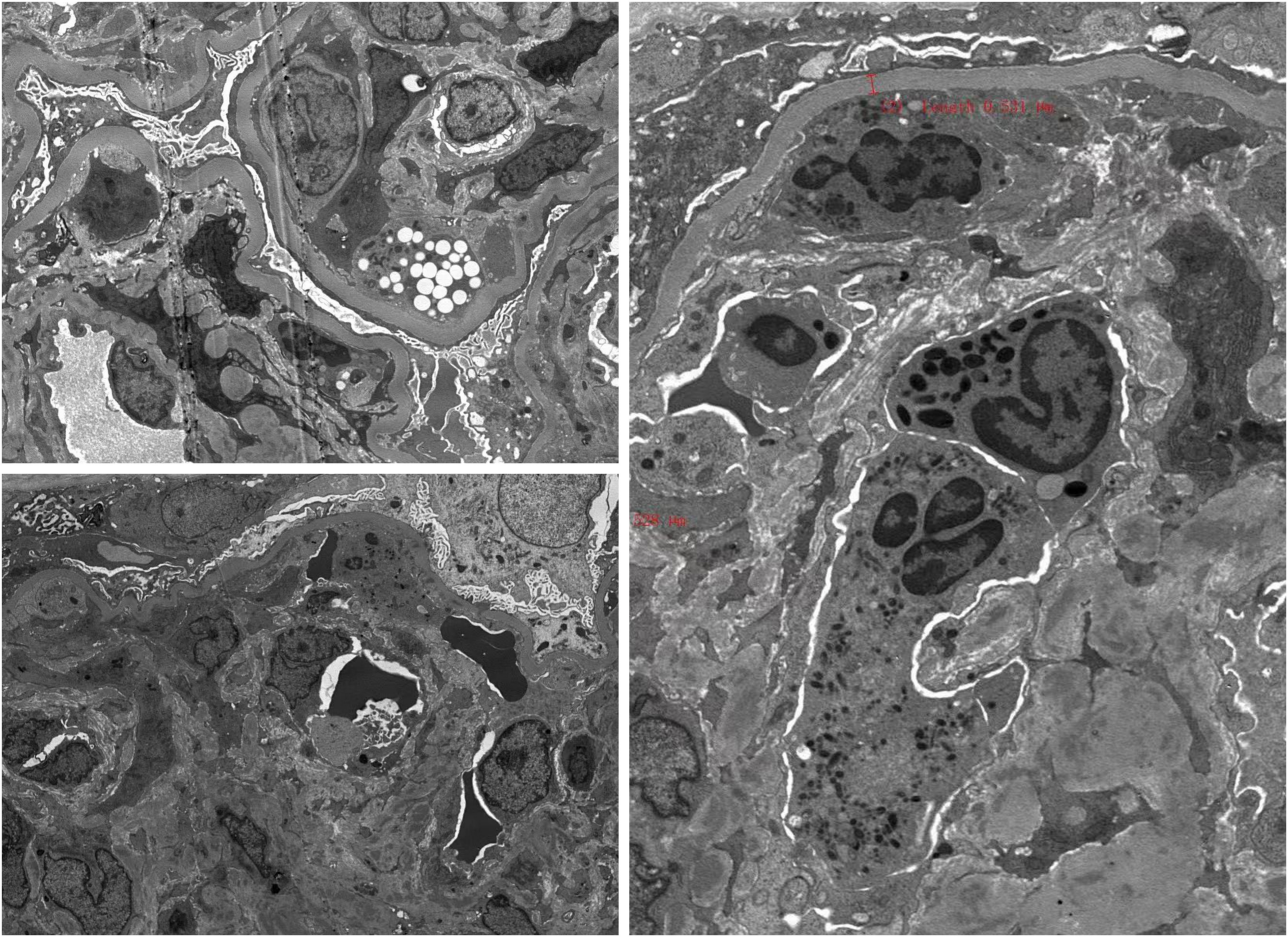
Electron microscopy shows segmental uniform thickening of the glomerular basement membrane, with segmental mesangial matrix interposition and double-contour (tram-track) formation. Diffuse podocyte foot process effacement. Segmental subepithelial hump-like electron-dense deposits are present, along with electron-dense deposits in the subendothelial and mesangial areas.

Light microscopic examination of 12 glomeruli revealed one with ischemic obsolescence. There was moderate to severe diffuse hyperplasia of mesangial cells and matrix, with diffuse glomerular basement membrane thickening and double contour formation. Immunofluorescence showed IgG (+++), IgA (+), IgM (+), C3 (+++), C1q (+), IgG1 (+++), IgG2 (−), IgG3 (+/−), IgG4 (−), Kappa (++), and Lambda (+/−) (Figures 5, 6). Electron microscopy demonstrated proliferation of glomerular capillary endothelial cells, segmental mesangial interposition with double contours, severe mesangial hypercellularity and matrix expansion, and segmental subepithelial hump-like electron-dense deposits. Electron-dense deposits were also observed in the subendothelial and mesangial areas, without discernible substructure (Figure 7). The findings were consistent with “Proliferative Glomerulonephritis with Monoclonal IgG1-κ Deposits (PGNMID),” although lymphoproliferative or plasmacytotic disorders required exclusion. Further serum and urine free light chain quantification yielded the following results: serum free kappa chain 177.6 mg/L ↑ (reference range: 3.3–19.4), serum free lambda chain 105.53 mg/L ↑ (5.71–26.3), κ/λ ratio 1.6829 ↑ (0.26–1.65); urine free kappa chain 456.34 mg/L ↑ (1.17–86.4), urine free lambda chain 168.51 mg/L ↑ (0.27–15.21), κ/λ ratio 2.7081 (1.83–14.26). No M-protein was detected in serum or urine. Bone marrow smear indicated a plasma cell proportion of 0.5%. Consequently, the patient was diagnosed with IgG1κ-PGNMID.

## Treatment and follow-up

**FIGURE 8:**
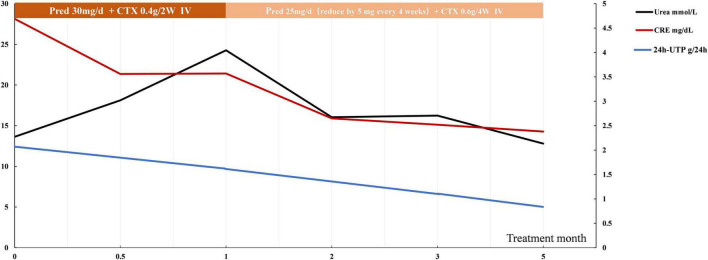
Timeline of glucocorticoid and cyclophosphamide therapy.

Following the diagnosis of IgG1-κ PGNMID, the patient was evaluated and found to have normal mental status and appetite, afebrile, with a repeat chest X-ray showing no significant exudative changes (Figure 3C). Serum and urine free light chain ratios (κ/λ) were within normal range. Although the bone marrow aspirate showed only 0.5% plasma cells, the 24-h urinary protein excretion remained above 3.5 g, and renal replacement therapy had already been initiated. Therefore, a regimen of prednisone plus cyclophosphamide was recommended as initial immunosuppressive therapy, with the understanding that the treatment plan would be adjusted based on follow-up assessments. Accordingly, one week after complete discontinuation of antibiotics, the patient was started on oral prednisone 30 mg daily and intravenous cyclophosphamide 0.4 g every two weeks. Concurrent medications included sacubitril/valsartan 100 mg twice daily orally (7–9), and benzathine penicillin 1.2 million units intramuscularly once monthly. After one month, the prednisone was tapered at a rate of 5 mg every four weeks, and the cyclophosphamide dose was adjusted to 0.6 g intravenously once monthly (Figure 8). At the 3-month follow-up, serum creatinine had decreased to 223 μmol/L; however, 24-h urinary protein excretion was 6.8 g/24 h (compared to 12.232 g/24 h during hospitalization). Repeat serum and urine free light chain analysis and a bone marrow biopsy were advised to guide further therapeutic adjustments. Nevertheless, the patient consistently declined further investigations and changes to the treatment regimen.

During follow-up until the fifth month, serum creatinine remained stable between 200 and 220 μmol/L, with a urine output of approximately 2,000 mL/24 h. The 24-h urinary protein excretion decreased to 1.987 g/24 h. Dialysis frequency was gradually reduced to once per week during this period. After six months of follow-up, an attempt was made to discontinue renal replacement therapy, and the patient was started on oral potassium-binding resin (sodium polystyrene sulfonate 5 g daily). However, subsequent follow-up became irregular. Nine months after discharge, we learned that the patient had been readmitted to a local hospital one month after stopping dialysis due to “bilateral lower limb edema, hyperkalemia, and upper respiratory tract infection.” The patient accepted the recommendation to resume hemodialysis but did not continue with prescribed medications. At the most recent outpatient follow-up, laboratory tests showed a hemoglobin level of 100 g/L, serum albumin of 26 g/L, urea of 16.47 mmol/L, and creatinine of 316 μmol/L. A 24-h urine collection was not provided. A repeat bone marrow smear showed no plasma cells. The patient’s family declined further serum and urine immunofixation electrophoresis and a repeat renal biopsy, insisting on continuation of hemodialysis therapy.

## Discussion

PGNMID is one type of monoclonal gammopathy of renal significance. It was initially put forward by Nasr et al. (10) from the Mayo Clinic in 2004. The deposition of monoclonal immunoglobulin within the glomeruli serves as the primary cause underlying renal manifestations. Specifically, the incidence of proteinuria can reach as high as 95%. Other clinical symptoms include non-specific manifestations like edema, hypertension, renal insufficiency, and hematuria. For patients with concurrent extrarenal diseases, the diagnosis becomes even more challenging. Diagnostic criteria: (1) Positive staining for immunoglobulin heavy chain (IgG), while negative staining for IgA and IgM heavy chains; (2) Positive staining for a single IgG subclass (IgG1/2/3/4); (3) Positive staining for a single light chain (kappa or lambda); (4) Under electron microscopy, there are mainly granular electron-dense deposits in the mesangium, subendothelial or subepithelial areas, similar to that seen in immune complex glomerulonephritis; (5) There is no clinical or laboratory evidence of cryoglobulinemia.

In PGNMID, immunofluorescence often reveals a predominant deposition of IgG3. This is primarily due to the propensity of IgG3 to self-aggregate via Fc segment interactions, forming large macromolecular complexes. This aggregation tendency facilitates its deposition within the kidney and promotes complement activation through the classical pathway. Furthermore, intrinsic glomerular cells, such as mesangial cells and endothelial cells, express various Fcγ receptors. Circulating monoclonal IgG3 can be specifically “captured” within the glomeruli through binding of its Fc portion to these receptors, leading to localized accumulation. With a molecular weight of approximately 170 kDa, IgG3 is a large immunoglobulin. Once retained in the glomeruli through the mechanisms described above—self-aggregation and Fcγ receptor binding—it becomes difficult to clear effectively. Its persistent presence contributes to ongoing glomerular injury (2, 11–13). The IgG1 subtype accounts for 24%–29% (14). Compared with patients of the IgG3, those with the IgG1 are more likely to be complicated by extrarenal diseases, and their clinical manifestations are often manifested as nephrotic syndrome. When performing standard serum protein electrophoresis (SPEP) combined with urine protein electrophoresis (UPEP) and immunofixation electrophoresis (IFE), 29.7% of patients may present with a monoclonal spike (M-spike) (14). Patients with the IgG1 subtype are more likely to have monoclonal immunoglobulin than those with the IgG3 subtype (25% versus 8%). A small number of patients who did not have M-spike detected during the initial diagnosis may show a positive result on IFE during subsequent follow-up examinations in the course of the disease. Patients with hypocomplementemia are more likely to have detectable monoclonal immunoglobulin and clones, which highlights the correlation between B- or plasma cell clones, monoclonal immunoglobulin and the complement system. Almost all patients have C3 deposits in the glomeruli, and 87% of them are accompanied by co-deposits of C1q. However, the intensity of C3 deposition in the IgG3 subtype is significantly higher than that in the IgG1, which is also the reason why patients with the IgG3 subtype are more likely to develop hypocomplementemia syndrome. The incidence of hypocomplementemia in adults is 20% (2). Among the histopathological types, 56.8% are membranoproliferative glomerulonephritis, 35.1% are endocapillary proliferative glomerulonephritis, and other types include mesangial proliferative glomerulonephritis and atypical membranous. In terms of histopathology, membranoproliferative glomerulonephritis is common in the IgG3 subtype, while both membranoproliferative glomerulonephritis and mesangial proliferative glomerulonephritis are seen in the IgG1 subtype. The patient in this study had a histopathological type of membranoproliferative glomerulonephritis. Obvious C3 deposits could be seen in the immunofluorescence, accompanied by hypocomplementemia. Meanwhile, it was the IgG1 subtype with a single κ deposition, which is quite rare.

Apart from disorders in complement regulation and immunoregulation, infection is also an important predisposing factor in the pathogenesis. PGNMID related to parvovirus B19 infection is extremely rare and has only been mentioned in case reports. All of these patients had a history of preceding infections and were characterized by proteinuria, edema, and hypoalbuminemia. Some patients also developed acute kidney injury. All three patients responded to treatment, and approximately two months after treatment, the renal abnormalities basically disappeared, indicating that the virus induced a transient IgG3 response, resulting in the accumulation of IgG3-κ in the glomeruli. It is thus speculated that PGNMID related to parvovirus B19 has self-limiting characteristics (15, 16). A study on pediatric showed that at least half of the patients had hypocomplementemia and infections before the occurrence of glomerulonephritis, mainly influenza B and group A streptococcal infection (17). Repeated infections may gradually amplify the abnormal alternative complement regulatory pathway and simultaneously induce the emergence of monoclonal immunoglobulin. Meanwhile, the concentration of monoclonal immunoglobulin in the circulation remains below the detection limit of routine tests such as immunofixation electrophoresis and protein electrophoresis. Eventually, the disease progresses to PGNMID (3). The immune response to exogenous antigens induced by infection may participate in the development of PGNMID through mechanisms related to B cell repertoire remodeling. It stimulates the differentiation of polyclonal B cells into plasma cells, which may also lead to the transformation of pathological types as the disease progresses (18). Our patient had repeated infections accompanied by hypocomplementemia within three months before the onset of the disease. After being admitted to the hospital, the antistreptolysin O was found to be significantly elevated, suggesting a possible streptococcal infection. It can be seen that in addition to disorders in complement and immunoregulation, infection is also an important aspect in the occurrence of this disease.

An important differential diagnostic consideration in our case was infection-related glomerulonephritis (IRGN), given the history of recurrent infections, markedly reduced serum C3, elevated anti-streptolysin O titer, and subepithelial hump-like electron-dense deposits. Renal biopsy, however, did not support a purely infection-related process. Immunofluorescence showed IgG1 subclass restriction and kappa light chain predominance, supporting PGNMID. Infection may have acted as a trigger but could not fully explain the renal lesion. C3 glomerulopathy was also considered because of hypocomplementemia, strong glomerular C3 staining, and an MPGN pattern. However, the presence of IgG1-kappa-restricted monoclonal deposits favored PGNMID over C3 glomerulopathy. Lupus nephritis and other immune complex-mediated glomerulonephritides should likewise be considered. Azhary et al. (19) reported a patient with preceding infection, low C3, nephrotic-range proteinuria, and an MPGN-like pattern who was ultimately diagnosed with lupus nephritis based on positive ANA by IFA and a full-house immunostaining pattern. In contrast, ANA, anti-dsDNA, and anti-ENA antibodies were negative here, and renal biopsy showed IgG1-kappa-restricted monoclonal deposits rather than a full-house pattern. Taken together, these findings support PGNMID rather than IRGN or lupus nephritis. This comparison highlights that in patients with antecedent infection and MPGN-like lesions, careful integration of serology, complement profile, and renal immunopathology is essential to distinguish PGNMID from infection-related glomerulonephritis and lupus nephritis.

Currently, there is no unified standard for the treatment of PGNMID. ACEI/ARB (angiotensin-converting enzyme inhibitors/angiotensin receptor blockers), glucocorticoids and immunosuppressants such as cyclophosphamide are relatively commonly used drugs. In a retrospective study conducted by Liu et al. (3), it was found that in the group receiving cyclophosphamide combined with prednisone, the complete remission (CR) rate was 11.1% and the overall response rate was 38.9%. In the group receiving bortezomib-based chemotherapy, the CR rate was 7% and the overall response rate was 71.4% (15). Moreover, the number of patients who developed end-stage renal disease was much lower than that in the group receiving cyclophosphamide combined with prednisone. Currently, more and more evidence suggests that compared with traditional immunotherapy and conservative treatment, targeted therapies targeting abnormal clones, such as rituximab and daratumumab, have more advantages in improving renal outcomes (20–22). For patients who relapse after bortezomib treatment, daratumumab combined with prednisone is also a potential option. For patients who relapse after bortezomib treatment, daratumumab combined with prednisone is also a potential option. However, PGNMID patients have a relatively low incidence of paraproteinemia and a low detection rate of abnormal clones. The detection rate of M–protein in urine and blood immunofixation electrophoresis is approximately 11.1–29%, and the detection rate of B or plasma cell clones in bone marrow is about 22.2%. This means that only a small proportion of patients can choose targeted therapy. IgM–type is more secreted by B cells. Therefore, if treatment for IgM–type PGNMID is initiated, drugs targeting B cells usually need to be included. Novel proximal complement inhibitors, such as iptacopan targeting factor B, can selectively inhibit the alternative pathway C3 convertase (C3bBb) and have been shown to reduce C3 deposition and ameliorate renal injury in models of C3 glomerulopathy (23, 24). A similar mechanism may also be applicable to PGNMID, in which monoclonal immunoglobulins trigger activation of the alternative and/or lectin pathways (25). However, further clinical studies are still needed to identify appropriate candidates through *in situ* convertase testing and to validate the efficacy and safety of this strategy.

## Data Availability

The original contributions presented in this study are included in this article/supplementary material, further inquiries can be directed to the corresponding authors.
